# Predictive Model of Paraaortic Lymph Node Involvement in cN0 Locally Advanced Cervical Cancers: PET/CT Technology Matters

**DOI:** 10.3390/diagnostics14222607

**Published:** 2024-11-20

**Authors:** Judicael Hotton, Emilie Raimond, Fabien Reyal, Sophie Michel, Vivien Ceccato, Abdenasser Moubtakir, Dimitri Papathanassiou, David Morland

**Affiliations:** 1 Department of Surgical Oncology, Institut Godinot, 51100 Reims, France; fabien.reyal@reims.unicancer.fr (F.R.); sophie.michel@reims.unicancer.fr (S.M.); vivien.ceccato@reims.unicancer.fr (V.C.); 2 CReSTIC, UR 3804, Université de Reims Champagne-Ardenne, 51687 Reims, France; dimitri.papathanassiou@reims.unicancer.fr (D.P.); david.morland@reims.unicancer.fr (D.M.); 3 Department of Obstetrics and Gynecology, CHU, 51100 Reims, France; eraimond@chu-reims.fr; 4 Department of Nuclear Medicine, Institut Godinot, 51100 Reims, France; abdenasser.moubtakir@reims.unicancer.fr

**Keywords:** locally advanced cervical cancer, ^18^F-FDG PET/CT, paraaortic lymph node involvement, time-to-flight technology, predictive PET/CT model

## Abstract

**Background**: The aim is to propose a model for predicting occult paraaortic lymph node (PALN) involvement in locally advanced cervical cancer (LACC) patients by including parameters such as reconstruction detection technology (use of time-of-flight) and parameters related to the primary tumor. This model will then be compared with the scores used in routine clinical practice; **Methods**: This retrospective observational cohort study included patients diagnosed with LACC who underwent ^18^F-FDG PET/CT prior to PALN surgical staging between February 2012 and May 2020. The following parameters were collected on PET/CT: tumor SUVmax, tumor MTV, number of common and distal pelvic node involvements. A multivariate regression analysis estimating the probability of PALN involvement was performed, with optimal thresholds determined via ROC curves; **Results**: In total, 71 patients met the inclusion criteria. Occult PALN involvement was detected in 12.7% of patients. A derived multivariate PET model selected four variables: number of common and distal iliac lymph nodes (OR 5.9 and 2.7, respectively), tumor-to-liver SUV ratio (OR 0.9) and the use of time-of-flight technology (OR 21.4 if no time-of-flight available). At the optimal threshold, a sensitivity of 77.8% and specificity of 88.7% was found. The model’s performances varied significantly between patients whose PET/CT used time-of-flight and those whose PET/CT did not. No significant differences were found between our model and the one used in clinical practice (*p* = 0.55); **Conclusions**: This study shows that PET/CT technology influences the ability to detect occult PALN involvement in LACC. This parameter should be considered in the regular revision of PET-based scores.

## 1. Introduction

Cervical cancer is a major global health problem, ranking fourth among the most common cancers in women worldwide and one of the leading causes of cancer-related death [1]. While early-stage cervical cancer is often curable, a significant challenge remains in managing patients diagnosed at advanced stages. Nearly 50% of patients present with locally advanced cervical cancer (LACC) [2], corresponding to stages IB3 to IVA according to FIGO classification [2]. LACC is characterized by tumor extension beyond the cervix, often involving parametrial, vaginal, or pelvic structures, and may also include regional lymph node involvement. The standard treatment approach for LACC includes a combination of pelvic radiotherapy and concomitant low-dose platinum-based chemotherapy, followed by brachytherapy [3,4]. This treatment scheme aims to achieve local control and prevent recurrence; however, despite its efficacy, the prognosis for patients with LACC remains low. The 5-year overall survival (OS) rates hover around 70%, but distant relapse is a frequent occurrence [5]. Among the prognostic factors influencing outcomes in LACC, paraaortic lymph node (PALN) status stands out as one of the most critical. PALN involvement is strongly associated with distant metastasis and significantly reduced survival [6], with the 5-year OS for patients with confirmed PALN metastases being less than 40%. For patients with PALN involvement, the risk of distant failure is notably higher [7,8]. The accurate detection of PALN metastasis is, therefore, essential to guide treatment planning, as it determines the need for extended radiation fields and can justify treatment intensification [9]. While conventional imaging techniques like MRI and CT can suggest lymph node involvement, they are often limited by their inability to detect occult metastases, which can lead to the underestimation of disease spread.

Laparoscopic surgical aortic staging, a mini-invasive technique, offers a method to more accurately assess PALN status [10]. By tailoring the radiotherapy field based on surgical findings, this approach reduces the risk of both undertreatment and unnecessary extended field radiation, potentially improving outcomes for patients. However, laparoscopic staging comes with its own set of challenges, including the potential for increased postoperative morbidity and delays in initiating definitive chemoradiotherapy [11]. To mitigate these drawbacks, ^18^F-FDG PET/CT was thus introduced as a surrogate technique for surgical PALN staging [3,12]. PET/CT allows for the visualization of metabolic activity in lymph nodes, with high ^18^F-FDG uptake being a strong indicator of malignancy. Nevertheless, despite its utility, PET/CT is not without limitations. False-negative rates for PALN detection range from 6 to 15% [13,14,15], primarily due to the limited spatial resolution of the technique, which can result in missed small or early metastatic deposits [16]. Various predictive models and scores have been proposed to reduce false negatives. One of the most widely used in clinical practice is the RT score, which evaluates more distal lymph nodes to infer PALN involvement [17,18]. According to this score, if no PALN uptake is detected on PET/CT, PALN involvement can still be presumed if certain criteria are met, such as the presence of at least one common iliac lymph node or three distal iliac lymph nodes showing ^18^F-FDG uptake.

However, this score does not consider the technological evolution that PET acquisition systems have undergone. Among these, time-of-flight technology (TOF) has been one of the most important technological breakthroughs, with a major impact on diagnostic performances. TOF results in an improved trade-off between lesion contrast, image noise, and total imaging time, leading to a combination of improved lesion detectability, reduced scan time or injected dose, and more accurate and precise lesion uptake measurement [19,20]. Given these technological advancements, it is becoming increasingly apparent that the current RT score may be insufficient for detecting occult PALN metastasis in its entirety. Other PET/CT-derived parameters, such as metabolic tumor volume (MTV), could offer additional prognostic value, as they provide more comprehensive data on tumor burden and metabolic activity. Incorporating these factors into a predictive model could potentially lead to more accurate staging and treatment personalization for patients with LACC.

The aim of this study is to build and evaluate a PET-based model for predicting occult PALN involvement in locally advanced cervical cancer. This model will incorporate overlooked factors such as TOF technology and PET-derived MTV to improve diagnostic accuracy. A secondary objective of this study will be to compare this new model to the currently used RT score, assessing its relative effectiveness in predicting PALN metastasis. By enhancing our understanding of PALN involvement through advanced imaging techniques, we hope to improve treatment planning and outcomes for patients with LACC.

## 2. Materials and Methods

### 2.1. Study Population

A retrospective observational bicentric cohort study was conducted. All patients treated for LACC who underwent ^18^F-FDG PET/CT prior to PALN surgical staging were screened from February 2012 to May 2020. Inclusion criteria were as follows: histological diagnosis of cervical carcinoma; locally advanced disease according to FIGO classification [2]; no PALN involvement based on pelvic MRI and ^18^F-FDG PET/CT imaging. Exclusion criteria were as follows: ^18^F-FDG PET/CT DICOM files unavailable, staging after radical hysterectomy for known cervical carcinoma, paraaortic or distant metastasis on pre-therapeutic PET/CT staging, concomitant or previous carcinoma.

### 2.2. Paraaortic Lymph Node Dissection Technique

The technique for laparoscopic retroperitoneal PALN lymphadenectomy in patients with LACC involves several key steps. The procedure begins with transperitoneal laparoscopy to assess for occult peritoneal carcinomatosis, which, if identified, leads to exclusion from surgery. If no carcinomatosis is present, the extraperitoneal approach is initiated through an incision near the left iliac spine, and blunt dissection is used to create the extraperitoneal space. Key anatomical landmarks such as the psoas muscle, ureter, and common iliac artery are identified, and CO_2_ insufflation is used to maintain the working space. Lymph node dissection is performed along the common iliac arteries, inferior mesenteric artery, aorta, until the inferior edge of left renal vein [21]. The procedure is concluded with marsupialization of the peritoneum to reduce postoperative lymphoceles [22]. In this study, the extraperitoneal approach was the only technique used for PALN lymphadenectomy. The surgery was followed by the concurrent chemoradiotherapy and uterovaginal brachytherapy. Extended paraaortic fields irradiation up to the level of left renal vessels was performed in the event of PALN involvement [23].

### 2.3. ^18^F-FDG PET/CT

All examinations were performed according to European Association of Nuclear Medicine (EANM) procedure guidelines for tumor imaging [24]. At least 6 h of fasting were required prior to ^18^F-FDG administration. Administered ^18^F-FDG activity ranged from 3 to 4 MBq/kg. PET examinations were acquired in several different centers but were all centrally reviewed by 2 experienced nuclear medicine physicians (DM, AM). In cases of discrepancy between readers in categorizing pelvic lymph nodes as FDG-avid, a consensus was reached through joint review by the two nuclear medicine physicians to ensure consistent interpretation. All semi-quantitative and volumetric PET parameters were derived by both nuclear medicine physicians to maintain measurement consistency. To minimize inclusion of non-tumoral FDG uptake from adjacent urinary bladder or bowel, a thresholding technique or manual delineation were applied during PET tumor volume estimation. The exact PET model and reconstruction algorithms was not systematically available, but whether or not time-of-flight (TOF) technology was used in image reconstruction was known based on the acquisition date and questioning of the centers.

### 2.4. Data Collection

Data collected from each patient’s medical record included sociodemographic characteristics (age at diagnosis), radiological data (MRI tumor size, TNM status, FIGO classification), histology. PET/CT parameters were extracted using a 41% threshold region of interest (ROI) encompassing the primary tumor lesion. Collected parameters were as follows: tumor SUVmax, tumor SUVmean, MTV, TLG, number of pelvic nodes (common iliac and/or distal external iliac FDG-avid nodes). Tumor/liver ratios (later denoted as T/L ratios) were calculated as a way to mitigate intermachine variability using the tumor SUVmax and the liver SUVmean. Liver SUVmean was measured using a spherical volume of interest positioned in the right lobe. Measures were performed using Syngo.via software version VB40A (Syngo.via, Siemens Healthineers, Erlangen, Germany).

### 2.5. Ethics

Data management was performed in accordance with current French regulations, in particular the General Data Protection Regulation 2016/679 of the European Parliament and of the Council of 27 April 2016, applicable since 25 May 2018 (RGPD), as well as the Data Protection Act of 6 January 1978, as amended in 2018. The database was created in accordance with CNIL reference method MR004 (n° F20230619164536, date: 1 June 2023). As the study was purely observational and based on an analysis of anonymized retrospective data, it was exempt from Institutional Review Board approval according to the French Public Health Code (L1121-1, law 2012–300, 5 March 2012). Informed consent was obtained from all subjects involved in this study. Patient data were de-identified before any statistical analysis.

### 2.6. Statistical Analysis

#### 2.6.1. Model Construction

Continuous variables were described by their mean and standard deviation. Categorical variables were described as numbers and percentages. Comparisons between PALN+ group and PALN− group, as defined at histological analysis, were conducted using Wilcoxon or Fisher’s exact test when appropriate.

Univariate analyses were conducted for PALN positivity prediction using logistic regression. Results are presented as odd ratios (OR) and 95% confidence intervals (95%CI). T/L ratio was preferred over SUVmax and SUVmean to avoid intermachine variability. TLG was not included for multicollinearity issues as it is the product of SUVmax (hence linked to T/L ratio) and MTV (which was already included).

Multivariate analysis was conducted using a model selection procedure based on Akaike criteria. The derived model provides a probability of PALN involvement. Receiver Operating Characteristic (ROC) curves were used to find an optimal threshold.

#### 2.6.2. Model Comparison

The optimal multivariate model (PET model) was compared with the RT score in which PALN is considered involved if at least one common iliac lymph node is noted or 3 distal iliac lymph nodes [17,18]. This score is used in clinical practice to decide paraaortic irradiation in N0 patients. Sensitivity and specificity of both scores were calculated along their 95%CI and compared using a McNemar test. Impact of TOF usage on both models was calculated using Fisher’s exact test. A *p*-value of less than 0.05 was considered significant.

## 3. Results

A total of 83 patients were identified, including 80 patients without PALN involvement on PET/CT. Nine patients were excluded because of unavailable PET/CT images. Thus 71 patients without PALN involvement on PET/CT were included. Clinicopathological characteristics are detailed in Table 1. The mean age at diagnosis was 51.4 years. The mean tumor size was 52.0 mm. The majority were squamous cell carcinomas (85.9%). Parametrial involvement was described in 32 (45.1%) cases.

Occult PALN involvement (PALN+) was seen in 9/71 patients (12.7%). TOF imaging was used for 56/71 (78.9%) of patients. In the PALN+ group, 5/9 (55.6%) had TOF imaging, compared with 51/62 (82.3%) in the PALN− group, although this difference was not statistically significant (*p* = 0.09). The primary tumor had a mean SUVmax of 15.4, with a T/L ratio of 7.1. SUV parameters did not significantly differ between PALN+ and PALN− groups (Table 2). MTV was significantly higher in PALN+ group (53.4 mL versus 41.6 mL, *p* = 0.046). A higher number of common iliac and distal iliac lymph nodes were described in the PALN+ group.

At univariate analysis, the number of positive iliac lymph nodes were associated with a stronger risk of PALN involvement, whether common iliac or distal (OR 4.6 *p* = 0.004 and 1.9 *p* = 0.041, respectively). TOF usage was not significant (*p* = 0.08). The optimal multivariate model retained four variables: number of common iliac lymph nodes, number of distal iliac lymph nodes, T/L ratio, and TOF usage. The results are presented in Table 3.

**Figure 1:**
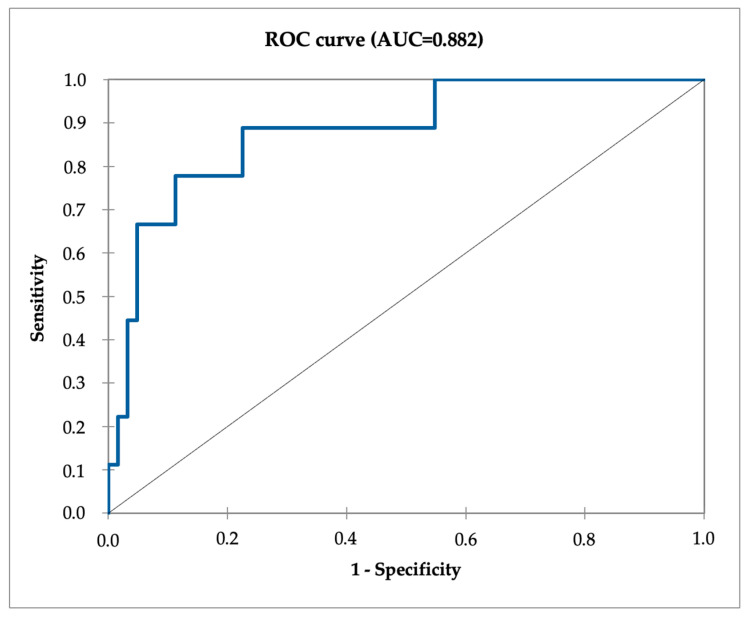
ROC curve illustrating the diagnostic performance of the PET model for predicting occult PALN involvement, with an AUC of 0.882.

Each positive common iliac lymph node on PET/CT increased the risk of occult PALN involvement (OR 5.85, *p* = 0.01), as well as each distal iliac lymph node (OR 2.71, *p* = 0.03). Non-TOF PET/CT systems had a significantly higher chance of missing PALN involvement (OR 21.4, *p* = 0.02). Although non-significant, the T/L ratio improved the model performance (OR 0.89, *p* = 0.56). This multivariate model gives a probability of PALN involvement. The optimal threshold to predict PALN involvement was >21.5% based on ROC curve analysis with an area under the curve of 0.882 (Figure 1).

Using this threshold, our model achieved a sensitivity of 77.8% [44.1–94.3] and a specificity of 88.7% [78–94.6], resulting in two false negative (Table 4). Performances were significantly different between patients who underwent PET on non-TOF PET/CT systems and TOF PET/CT systems (*p* = 0.02).

Using the RT score, sensitivity was 55.5% [26.7–80.9] and specificity 87.0% [76.1–93.4], resulting in four false negatives (Table 4). Performances were again significantly different between patients who underwent PET on non-TOF PET/CT systems and TOF PET/CT systems (*p* = 0.035). However, no significant discrepancies between RT and PET models were found (*p* = 0.55).

## 4. Discussion

In recent years, ^18^F-FDG PET/CT has gradually replaced surgical staging in the evaluation of PALN involvement in LACC. However, despite its advancements, this imaging modality is not without limitations; false negative results have been described in 10–15% of cases, which raises significant concerns regarding the potential for the under-treatment of patients [25]. This risk underscores the necessity for more reliable predictive models that can enhance diagnostic accuracy and treatment planning. To address these challenges, various predictive models have been developed. Yet, many of these models have failed to adequately account for the inherent heterogeneity present within the PET/CT fleet, leading to variable results across different imaging systems. Among the most widely utilized predictive frameworks is the model derived from the study by Lee et al. [17]. This model states that occult PALN involvement should be suspected when there is at least one common iliac lymph node or three or more distal iliac lymph nodes.

The PET model we have developed in this article reflects the importance of the number of iliac lymph nodes and their proximal nature in the prediction of occult PALN involvement. Our analysis reveals that each positive common iliac lymph node identified on PET/CT significantly increases the risk of occult PALN involvement, with an OR of 5.85. This contrasts with the lower risk associated with more distal iliac lymph nodes, which demonstrated an OR of only 2.71. These findings align with the existing literature: in patients exhibiting no uptake in pelvic lymph nodes on ^18^F-FDG PET/CT, the risk of undetected PALN metastasis remains under 5% [14,26,27]. However, when pelvic lymph nodes demonstrate high metabolic activity, the prevalence of undetected PALN metastasis can exceed 50% [14,27].

Furthermore, our study revealed that non-TOF PET/CT systems present a significantly higher risk of overlooking PALN involvement, with an OR of 21.4. This finding is consistent with established knowledge that TOF technology markedly enhances sensitivity and lesion detection capabilities [28,29]. The improved spatial resolution and superior characterization of regions with low FDG uptake enable nuclear medicine physicians to identify smaller lesions more effectively. PET/CT systems equipped with TOF technology can detect small lesions ranging from 4 to 6 mm, a capability that is particularly relevant for identifying metastatic lymph nodes that may otherwise be missed [30]. As imaging technology continues to evolve, it is plausible that similar disparities in detection capabilities will emerge with the advent of new digital PET/CT. These scanners, while not represented in our current study population, may further enhance diagnostic accuracy and lead to improved patient outcomes. Our PET model demonstrated superior performance compared with a simple lymph node count on non-TOF machines, with a sensitivity of 100% versus 25% with the conventional RT score.

In addition to TOF technology, advances such as long axial field-of-view (LAFOV) PET/CT offer enhanced sensitivity, faster imaging, and the ability for low-dose protocols, which could further revolutionize oncological imaging by improving diagnostic precision and expanding clinical applications [31,32].

Tumor FDG uptake was included in the final model with the T/L ratio. Although the impact of this variable was non-significant in isolation, the model exhibited enhanced performance when incorporating this parameter. Interestingly, the odds ratio for the T/L ratio was less than 1, suggesting that primary tumors characterized by low FDG uptake are more likely to harbor occult PALN involvement. The observation of an odds ratio below 1 for the T/L ratio suggests that primary tumors with lower FDG uptake may have a higher likelihood of occult PALN involvement [33]. This finding may be influenced by biological factors such as variations in tumor metabolic activity or hypoxia within the tumor microenvironment, which could impact both FDG uptake levels and metastatic potential. Initially, MTV appeared promising in our analysis, revealing a significantly higher value in cases of PALN involvement (53.4 mL versus 41.6 mL, *p* = 0.046). However, this variable was ultimately excluded from the multivariate analysis.

Despite these promising findings, our model’s performance did not yield a statistically significant improvement over an RT score. This lack of distinction may be attributed to the uneven distribution of PET/CT technologies, particularly between TOF and non-TOF systems. This study has limitations: its retrospective design and the relatively small sample size inherently constrain the robustness of our conclusions. Although adjustments were made for the variability in PET/CT technology, these findings highlight the necessity of standardized imaging protocols to reduce potential biases and improve predictive accuracy across diverse clinical settings. While all PET/CTs were centrally reviewed to ensure consistency, the variations in imaging protocols and equipment across different centers introduced additional heterogeneity into the data. Moreover, the retrospective nature of the study did not allow for the standardization of PET scanner models or reconstruction algorithms, which may impact the derived imaging parameters and should be considered when interpreting results. Future prospective studies are warranted to address these limitations and to refine the predictive capabilities of our model in diverse clinical settings.

## 5. Conclusions

This study shows that PET/CT technology evolution influences the ability to detect occult PALN involvement in LACC. This parameter should be taken into account in the regular revision of PET-based scores. Iliac lymph node involvement is a major predictor of PALN involvement, especially if it is proximal. An analysis of the uptake of the primary tumor could be of added interest. Future prospective multicenter studies with standardized PET/CT protocols are warranted to validate the model and confirm its utility across diverse clinical settings, thereby enhancing its generalizability and reliability in guiding treatment planning.

## Figures and Tables

**Table 1:** Clinicopathological characteristics of the population.

	Whole Population n = 71
Age in years, mean (sd)	51.4 (11.2)
Tumor size in mm, mean (sd)	52.0 (15.6)
Histological type, n (%)	
Squamous cell carcinoma	61 (85.9%)
Adenocarcinoma	10 (14.1%)
TNM stage, n (%)	
IB3	14 (19.7%)
IIA	18 (25.4%)
IIB	32 (45.1%)
III	7 (9.9%)
FIGO stage 2018, n (%)	
IB3	3 (4.2%)
IIA	15 (21.1%)
IIB	19 (26.8%)
IIIA-B	2 (2.8%)
IIIC	32 (45.1%)

**Table 2:** Age and PET parameters along PALN involvement.

	Whole Population n = 71	PALN+ n = 9	PALN− n = 62	*p*-Value
Age in years, mean (sd)	51.4 (11.2)	54.0 (8.9)	51.0 (11.5)	0.26
FDG PET/CT parameters				
TOF imaging used, n (%)	56 (78.9%)	5 (55.6%)	51 (82.3%)	0.09
SUVmax, mean (sd)	15.4 (6.2)	14.2 (4.9)	15.5 (6.4)	0.47
SUVmean, mean (sd)	9.3 (3.9)	8.8 (3.0)	9.4 (4.1)	0.41
T/L ratio, mean (sd)	7.1 (3.2)	7.3 (2.9)	7.1 (3.2)	0.29
MTV mL, mean (sd)	43.1 (29.9)	53.4 (25.0)	41.6 (30.4)	0.046 *
TLG mL, mean (sd)	427.7 (349.5)	502.3 (293.7)	416.9 (357.6)	0.06
Positive LN, mean number (sd)				
Common iliac LN	0.2 (0.5)	0.8 (1.0)	0.1 (0.4)	<0.001 *
Distal iliac LN	0.8 (1.0)	1.4 (1.1)	0.7 (1.0)	<0.001 *

*: *p* < 0.05; T/L ratio, tumor/liver SUVmax ratio; LN: lymph nodes.

**Table 3:** Univariate and multivariate analysis (PET model).

	Univariate Analysis	Multivariate Analysis
Each positive common iliac lymph node	4.61 [1.61;13.18] *p* = 0.004	5.85 [1.44;23.80] *p* = 0.01
Each positive distal iliac lymph node	1.91 [1.03;3.57] *p* = 0.041	2.71 [1.08;6.77] *p* = 0.03
T/L ratio	1.02 [0.82;1.27] *p* = 0.84	0.89 [0.62;1.30] *p* = 0.56
MTV	1.02 [0.99;1.04] *p* = 0.27	Rejected
Non-TOF PET/CT system	3.71 [0.86;16.09] *p* = 0.08	21.4 [1.66;276.96] *p* = 0.02

T/L ratio, tumor/liver SUVmax ratio; TOF, time-to-flight.

**Table 4:** Comparison of diagnostic performances of PET model with existing RT model.

	Whole Population		Patients Who Performed PET/CT on a non-TOF System		Patients Who Performed PET/CT on a TOF System	
	PET Model	RT Model	PET Model	RT Model	PET Model	RT Model
Sensitivity						
Percentage	77.8%	55.5%	100%	25.0%	60.0%	80.0%
95%CI	[44.1–94.3]	[26.7–80.9]	[44.9–100]	[3.9–71]	[23.1–88.0]	[35.8–97.4]
Specificity						
Percentage	88.7%	87.0%	72.7%	100.0%	92.1%	84.3%
95%CI	[78.0–94.6]	[76.1–93.4]	[42.8–90.5]	[69.4–100]	[80.8–97.3]	[71.6–92.0]
True positive	7	5	4	1	3	4
True negative	55	54	8	11	47	43
False positive	7	8	3	0	4	8
False negative	2	4	0	3	2	1

TOF, time-to-flight; RT, radiotherapy.

## Data Availability

The original contributions presented in the study are included in the article, further inquiries can be directed to the corresponding author.
